# Climate-Endangered Arctic Epishelf Lake Harbors Viral Assemblages with Distinct Genetic Repertoires

**DOI:** 10.1128/aem.00228-22

**Published:** 2022-08-25

**Authors:** Myriam Labbé, Mary Thaler, Thomas M. Pitot, Josephine Z. Rapp, Warwick F. Vincent, Alexander I. Culley

**Affiliations:** a Département de Biochimie, de Microbiologie et de Bio-Informatique, Université Laval, Québec, Québec, Canada; b Institut de Biologie Intégrative et des Systèmes (IBIS), Université Laval, Québec, Québec, Canada; c Centre d’Études Nordiques (CEN), Université Laval, Québec, Québec, Canada; d Département de Biologie, Université Laval, Québec, Québec, Canada; University of Nebraska—Lincoln

**Keywords:** Arctic, *Caudovirales*, Last Ice Area, *Pelagibacter* phage, *Phycodnaviridae*, auxiliary metabolic genes, epishelf lake, viruses

## Abstract

Milne Fiord, located on the coastal margin of the Last Ice Area (LIA) in the High Arctic (82°N, Canada), harbors an epishelf lake, a rare type of ice-dependent ecosystem in which a layer of freshwater overlies marine water connected to the open ocean. This microbe-dominated ecosystem faces catastrophic change due to the deterioration of its ice environment related to warming temperatures. We produced the first assessment of viral abundance, diversity, and distribution in this vulnerable ecosystem and explored the niches available for viral taxa and the functional genes underlying their distribution. We found that the viral community in the freshwater layer was distinct from, and more diverse than, the community in the underlying seawater and contained a different set of putative auxiliary metabolic genes, including the sulfur starvation-linked gene *tauD* and the gene coding for patatin-like phospholipase. The halocline community resembled the freshwater more than the marine community, but harbored viral taxa unique to this layer. We observed distinct viral assemblages immediately below the halocline, at a depth that was associated with a peak of prasinophyte algae and the viral family *Phycodnaviridae.* We also assembled 15 complete circular genomes, including a putative *Pelagibacter* phage with a marine distribution. It appears that despite its isolated and precarious situation, the varied niches in this epishelf lake support a diverse viral community, highlighting the importance of characterizing underexplored microbiota in the Last Ice Area before these ecosystems undergo irreversible change.

**IMPORTANCE** Viruses are key to understanding polar aquatic ecosystems, which are dominated by microorganisms. However, studies of viral communities are challenging to interpret because the vast majority of viruses are known only from sequence fragments, and their taxonomy, hosts, and genetic repertoires are unknown. Our study establishes a basis for comparison that will advance understanding of viral ecology in diverse global environments, particularly in the High Arctic. Rising temperatures in this region mean that researchers have limited time remaining to understand the biodiversity and biogeochemical cycles of ice-dependent environments and the consequences of these rapid, irreversible changes. The case of the Milne Fiord epishelf lake has special urgency because of the rarity of this type of “floating lake” ecosystem and its location in the Last Ice Area, a region of thick sea ice with global importance for conservation efforts.

## INTRODUCTION

**FIG 1:**
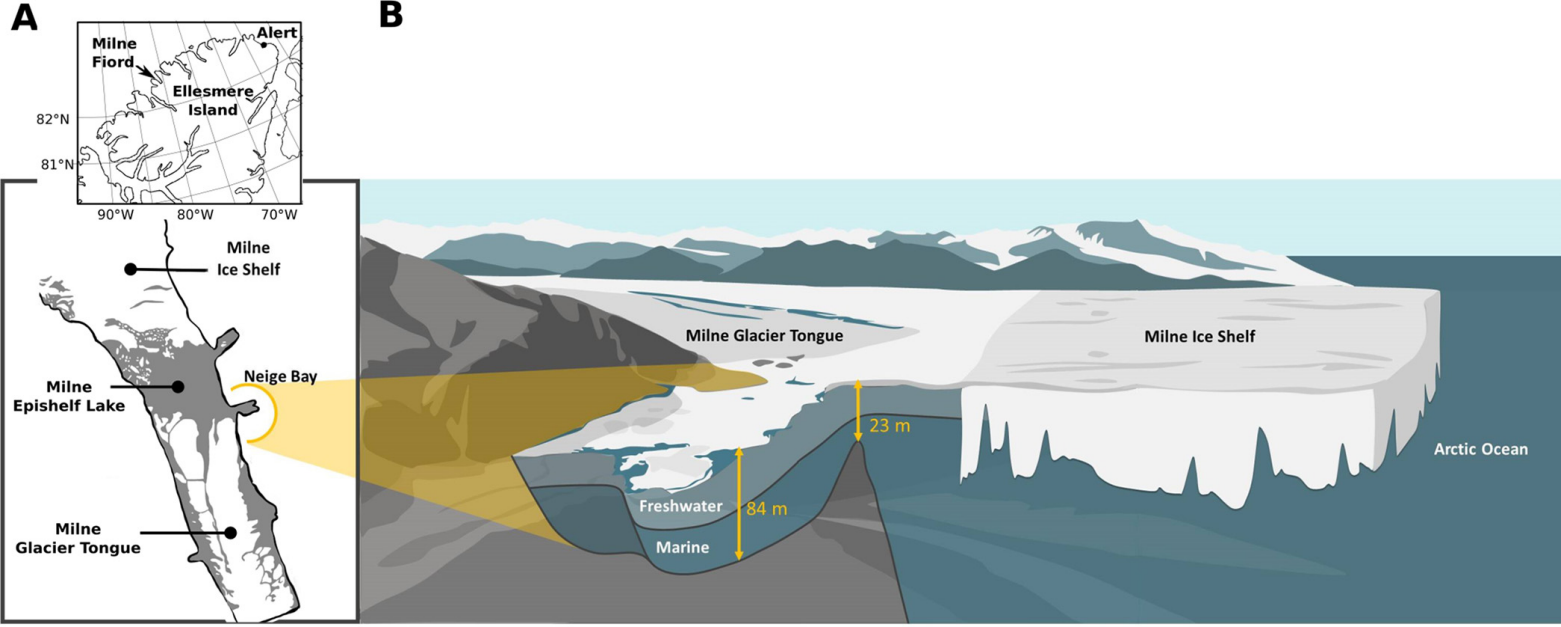
(A) General location and local geography of the Milne Fiord epishelf lake in 2015 (adapted from reference 7). Gray areas of map indicate lake ice detected by RADARSAT-2 imagery. (B) Cartoon showing accumulation of freshwater behind Milne Ice Shelf and the bottom topography of Neige Bay.

The cryosphere is changing rapidly throughout the world, particularly in the Arctic, where air temperatures are increasing at 2 to 3 times the global average (1). Sea ice cover is predicted to persist longest in the Last Ice Area (LIA), a region of thick ice north of Greenland and the Canadian Arctic Archipelago (2, 3), which contributes to persistent cold conditions for coastal habitats, including ice shelves, glaciers, and perennially ice-capped lakes. While the LIA is expected to be an ultimate refuge for many ice-dependent species, rapid warming threatens its diverse ecosystems, including epishelf lakes, which consist of freshwater impounded behind an ice shelf and retaining a hydraulic connection to the ocean. While historically a number of epishelf lakes were found in the Arctic (4), and such lakes are relatively common in Antarctica (5), Milne Fiord (82°35.54′N, 80°35.76′W, NU, Canada) is one of only two examples known in the Arctic; one other may be in eastern Greenland (6). In the Milne Fiord epishelf lake (MEL), meltwater runoff from an 1,108-km^2^ catchment area accumulates atop denser seawater behind an ice shelf across the mouth of Milne Fiord (7) (Fig. 1). The depth of stratification varies with freshwater inputs due to seasonal melt, with maximum depth corresponding to the minimum draft of the ice shelf (7). Ice-penetrating radar has determined that this minimum corresponds to a channel along the lower surface of the Milne Ice Shelf, draining freshwater into the Arctic Ocean (8). The lake’s existence thus depends on the integrity of the ice shelf, which has been in negative mass balance since the 1950s (9). Its vulnerability is illustrated by the drainage and loss of a similar epishelf lake in Disraeli Fiord following the breakup of the Ward Hunt Ice Shelf between 2000 and 2002 (10). In July 2020, a calving event reduced the Milne Ice Shelf area by about 43% and caused fractures in the remaining mass (11). Researchers have not yet determined whether these changes caused the irreversible loss of MEL (Derek Mueller, personal communication).

Previous studies of microbial diversity of MEL, using pigments, flow cytometry (FCM), and amplicon sequencing found distinct eukaryote and prokaryote communities in the freshwater and seawater layers (12, 13). While viruses are important components of aquatic ecosystems, exerting top-down control of their hosts through infection and lysis, they have received less attention. Diversity of T4-like bacteriophages was assessed by denaturing gradient gel electrophoresis, which showed strong structuring by stratification (12). Studies in other polar freshwater environments have found high viral diversity and abundance and suggest that viruses play a major role in bacterial dynamics and carbon cycling (14, 15). In an epishelf lake in Antarctica, viral dynamics were linked to bacterial production and the dissolved organic carbon pool (16). Viruses can also influence biogeochemical cycles by altering host cell functions. Their genomes can contain not only genes necessary for replicating and assembling virus particles, but also auxiliary metabolic genes (AMGs) that boost host metabolic functions and thus may enhance viral production (17). Our study is the first to apply shotgun metagenomic methods to the MEL viral community. We aimed to capture an in-depth snapshot of the extracellular community of double-stranded DNA (dsDNA) viruses in freshwater, halocline, and marine layers in summer 2016. We anticipated that these layers would have distinctive viral communities, as was observed in a highly stratified Arctic lake (18).

The metagenomic data presented from July 2016 are among the last obtained before the catastrophic calving event in 2020 and are a valuable record of an endangered LIA ecosystem whose loss is significant for global biodiversity.

## RESULTS

### Water column properties.

**FIG 2:**
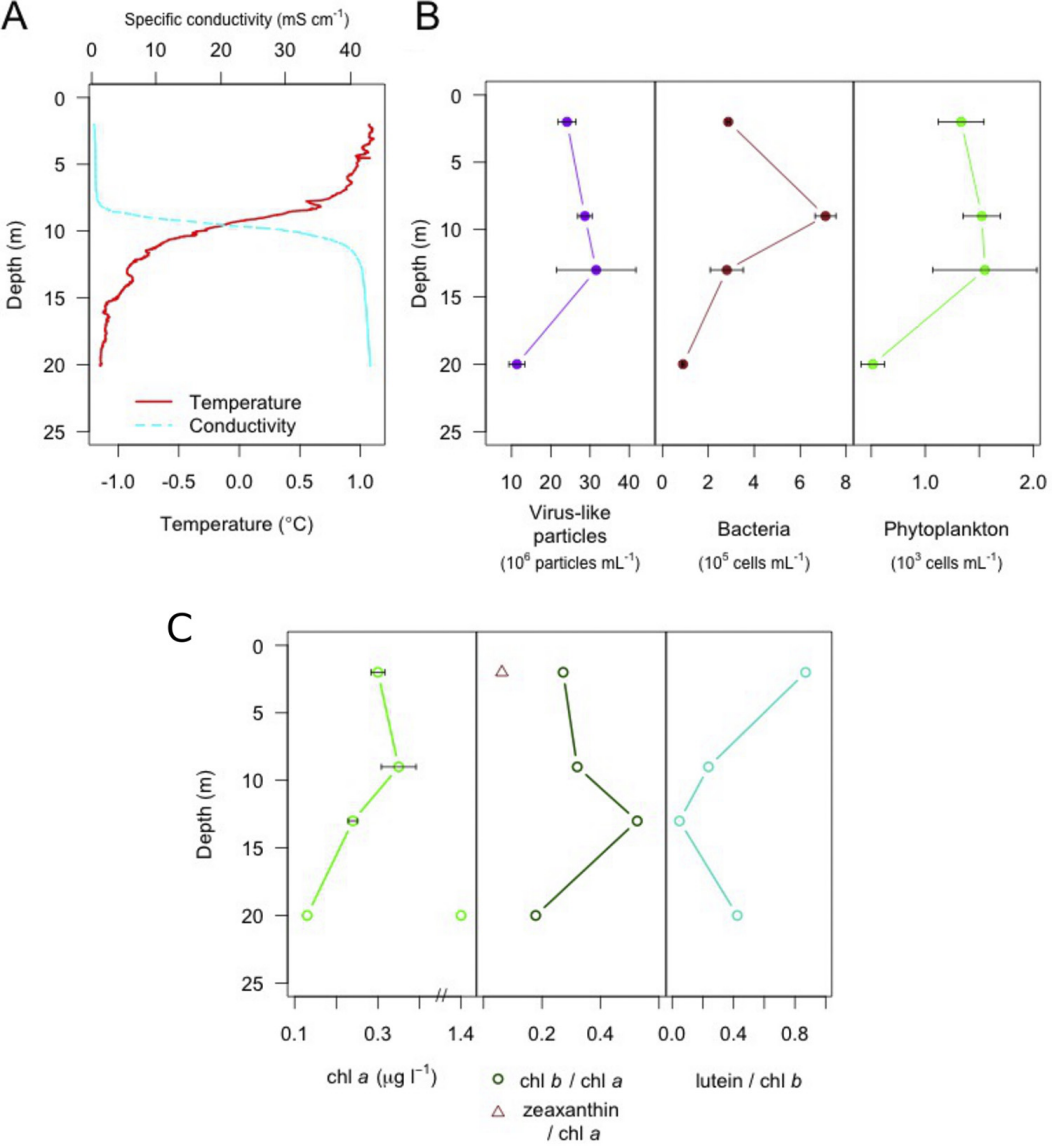
Water column profiles of the Milne Fiord epishelf lake. Shown are (A) physical properties measured using a YSI probe and CTD sensors, (B) virus-like particle and cell counts, obtained by flow cytometry, and (C) chlorophyll *a* concentrations and mass ratios of photosynthetic pigments in the water column determined by HPLC analysis. See the interpretation of phytoplankton taxonomy in Results. Error bars in panels B and C show standard error (*n* = 3).

We collected triplicate water samples from four depths (2, 9, 13, and 20 m) near the center of Neige Bay (unofficial name), which exchanges water with the rest of Milne Fiord over a 23-m sill (7) (Fig. 1). A stream flows into the bay’s eastern end. Our conductivity profile (Fig. 2A), supported by other water chemistry and nutrient data (see Table S1 in the supplemental material), shows the halocline at 8 m in 2016; thus, we consider samples from 9 m to be halocline and samples from 13 m to be lower halocline/marine. All physicochemical variables measured had a strong (>0.8) positive or negative Pearson correlation with depth, except for total phosphorus, whose correlation with depth was 0.64 (Table S1).

### Cellular community.

We place the viral community of MEL in the context of their potential hosts by characterizing the cellular community using FCM, accessory pigments, and small subunit rRNA (SSU rRNA) gene surveys. Picocyanobacterial cell concentrations detected by FCM were below the threshold of detection, comprising <0.5% of events, in contrast to previous years 2010 to 2012, when they were 1 to 10^3^ cells mL^−1^ (13). On the other hand, we cannot rule out the presence of filamentous cyanobacteria, which are not detected by FCM. For eukaryote picophytoplankton (henceforth “phytoplankton”), virus-like particles, and nonphotosynthetic prokaryotes (henceforth “bacteria”), the greatest variability of FCM counts between replicates occurred at 13 m, suggesting spatial heterogeneity at this depth (Fig. 2B). Due partly to this variability, significant difference by depth could only be detected for bacteria, whose abundance at 9 m was significantly higher than at 20 m (*P* < 0.05). The virus/prokaryote ratio at all depths was within an order of magnitude (range, 4 to 13), indicating no marked difference in overall viral activity.

We interpreted accessory pigment profiles as indicators of phytoplankton taxa (19). Zeaxanthin, which has been associated with picocyanobacteria in a High Arctic lake (20), was only detected at 2 m (Fig. 2C). Chlorophyll *b* (Chl *b*) was found throughout the water column, suggesting that green algae dominated, and the high lutein/Chl *b* ratio at most depths (Fig. 2C) indicated the presence of chlorophytes, possibly the previously detected *Radicarteria* clade (13). The exception was 13 m, where a low lutein/Chl *b* ratio, along with peaks in MgDVP and a micromonal-like pigment (data not shown), suggests it was dominated by prasinophyte algae, likely *Micromonas*, the most widespread picoprasinophyte taxon in the Arctic (21).

One 20-m replicate also had a chlorophyll *a* (Chl *a*) concentration 10 times higher than those of other replicates at this depth (Fig. 2C). The pigment profile resembled the other replicates at 20 m, suggesting the phytoplankton community in this sample was autochthonous, rather than transported to this depth. It might result from a colonial alga, such as *Phaeocystis*, or a chain-forming diatom.

Though samples were prefiltered to remove cells, we still detected SSU rRNA genes in our reads. These may represent free DNA from broken cells or cells of <0.22 μm. These data do not give a complete picture of the cellular community, but can confirm the presence of interesting taxa. 18S rRNA genes with hits to the prasinophyte *Micromonas* were detected at all depths except 2 m, and hits to the chlorophyte clade *Radicarteria* were detected at 2 and 9 m (Fig. S1). The Proteobacteria *Pelagibacter* clade (also called SAR11), a small, widespread marine bacterium (22) that could have passed through our prefiltration, was not detected at 2 m, but in the three deeper layers they comprised 7 to 19% of 16S rRNA genes (Fig. S2). The 9-m community was dominated by SAR11 clade III, while 13 and 20 m were dominated by clade I. Finally, cyanobacterial taxa were detected at low relative abundances at all depths, including the families *Nostocaceae*, *Phormidiaceae*, *Leptolyngbyaceae*, and *Cyanobiaceae*; their highest abundance was 0.6% of 16S rRNA genes at 2 m.

### Viral community.

**FIG 3:**
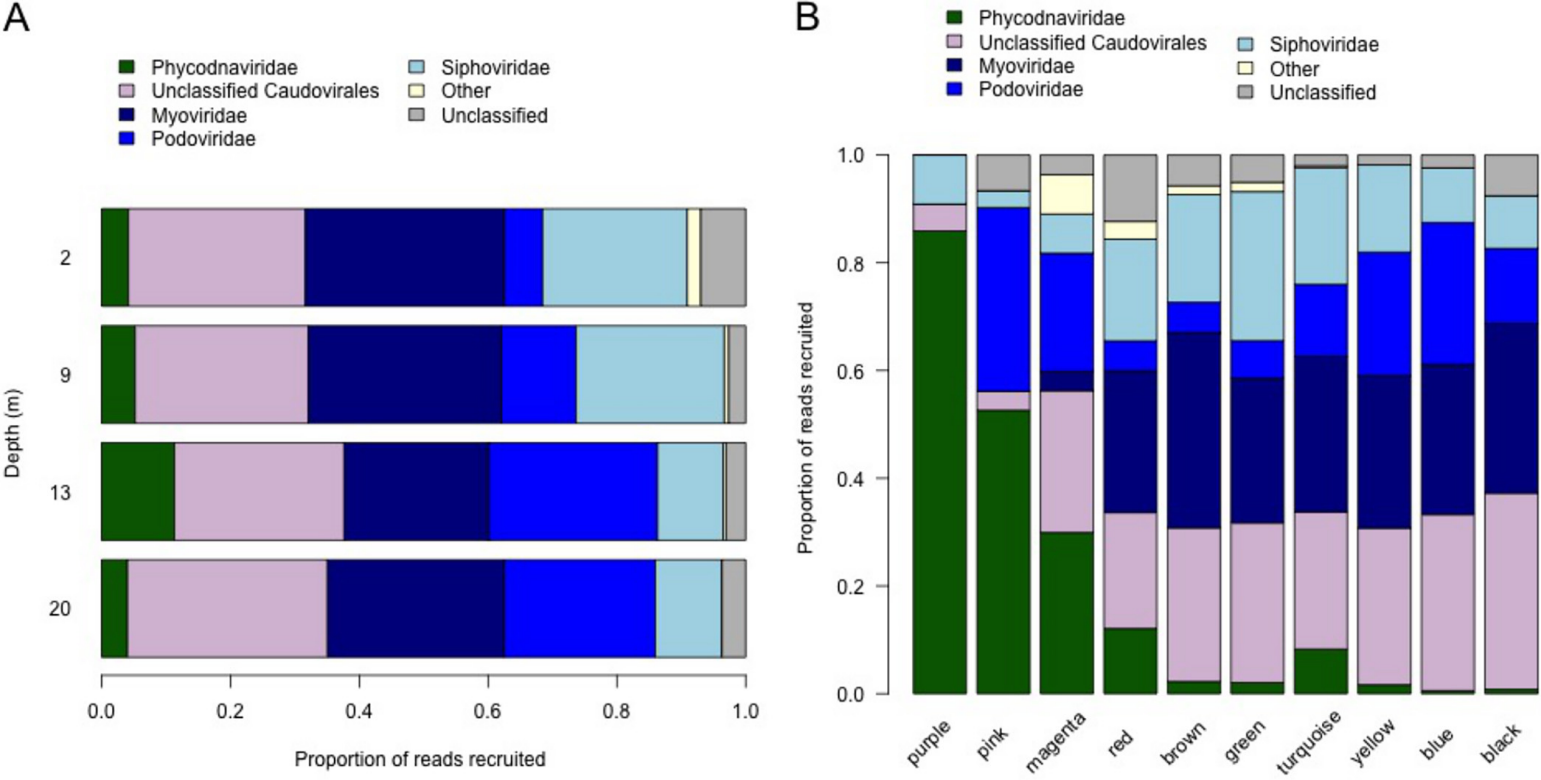
(A) Distribution of virus families by depth, as determined by VPF-Class. Relative abundance was calculated using the vOTU table standardized for contig sequence length. (B) Taxonomic composition of WGCNA modules.

We assessed viral diversity from DNA in the <0.22-μm fraction of the Milne Fiord water column, using a metagenomic approach that targeted dsDNA viruses (see Discussion for limitations of this methodology). These metagenomic reads yielded 2,290 viral operational taxonomic units (vOTUs), considered here as individual sequences representing a group of highly similar contigs. (The vOTU composition of the anomalous high-chlorophyll sample resembled other replicates at 20 m, so we proceeded to pool these samples for further analysis.) Most vOTUs were dsDNA bacteriophages, a group that is preferentially identified by the software we used to screen for viral sequences, VirSorter 1 (23). Traditionally placed in the order *Caudovirales*, with three major families, *Myoviridae*, *Podoviridae*, and *Siphoviridae*, dsDNA phage classification is currently undergoing major revision (24); however, we continue to use the older names for continuity with previous studies. *Podoviridae* was most abundant below the halocline, and *Siphoviridae* was most abundant above it (Fig. 3A). The only abundant (>1% of reads recruited) non-*Caudovirales* family was the nucleocytoplasmic large DNA virus (NCLDV) family *Phycodnaviridae*, with a peak at 13 m. *Phycodnaviridae* in our data set grouped into fewer, more abundant vOTUs at the 95% threshold (Fig. S3), indicating a lower diversity, possibly because larger phycodnaviruses were removed by the prefiltration step. A small number of vOTUs, 0.3% of viral reads recruited, were assigned to the family *Lavidaviridae*, also called virophages, which to date are known exclusively as coinfectants (sometimes called parasitic) on the NCLDV family *Mimiviridae* (25). Most (~75%) *Lavidaviridae* reads were found at 2 m.

**FIG 4:**
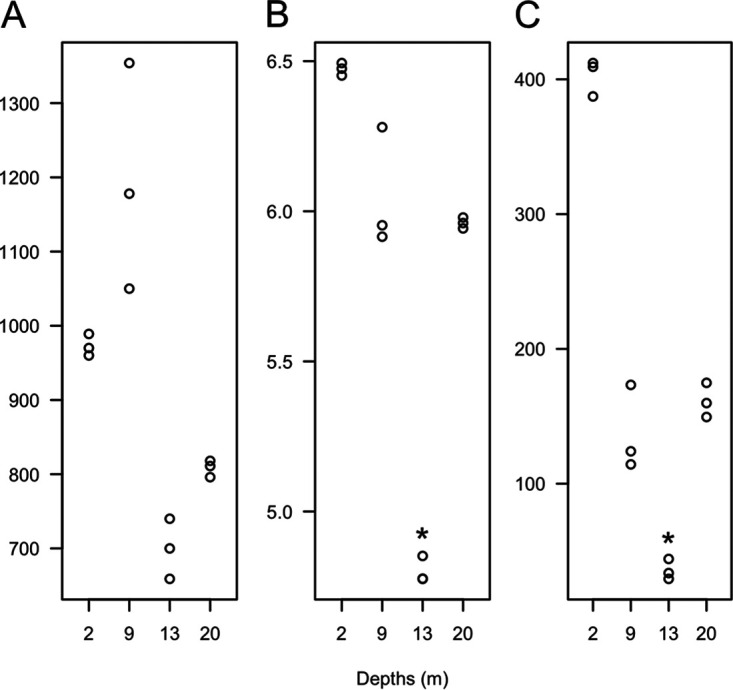
Alpha diversity calculated as (A) richness, (B) inverse Simpson index, and (C) Shannon index for vOTUs at different depths of the Milne Fiord epishelf lake. Values for triplicate independent samples are shown (*n* = 3). Asterisks indicate depths with significantly different diversity (*P* < 0.05).

**FIG 5:**
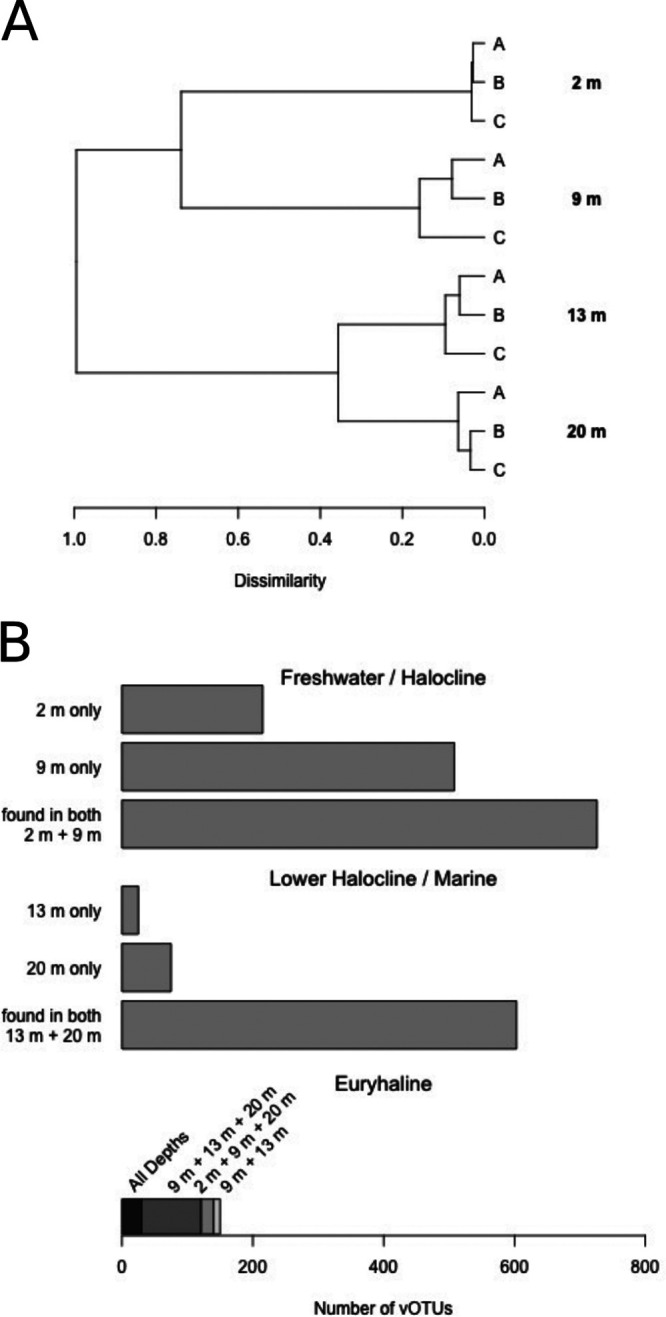
(A) Samples (three replicates per depth) clustered using Bray-Curtis distance on vOTU abundance data, normalized by sequence length and using Hellinger and log transformations; (B) number of vOTUs found at one depth only or at more than one depth. The bottom stacked bar groups vOTUs that are found both above and below the halocline, considered to be “euryhaline.”

Both richness and evenness drove differences in alpha diversity between depths (Fig. 4). Shannon and inverse Simpson indices were significantly lower in 13-m samples (*P* < 0.05) because a single vOTU, NODE170_03, comprised about 13% of all reads recruited at this depth. (Note that vOTU identifiers have no biological meaning; the word “node” originates from the read assembly step with MetaSPADes [26].) Clustering using Bray-Curtis distance grouped samples from the freshwater and halocline together and samples from the bottom of the halocline and marine layer (Fig. 5A). Samples which clustered together shared a greater proportion of vOTUs (Fig. 5B). Only 150 vOTUs, or 6.5%, were present in both clusters, and 47 of these belonged to *Phycodnaviridae*. The two shallower depths (2 m and 9 m) each had higher proportions of unique vOTUs than the two deeper depths (13 m and 20 m) (Fig. 5B).

To uncover the underlying structure of the viral community, we clustered virotypes into modules based on co-occurrence using weighted gene correlation network analysis (WGCNA), which has been used to delimit communities associated with ecosystem functions (27, 28). We consider that each module approximates a distinct set of related overlapping ecological niches. We also used automated and manual annotation to characterize the repertoire of virus-carried genes, including AMGs.

**FIG 6:**
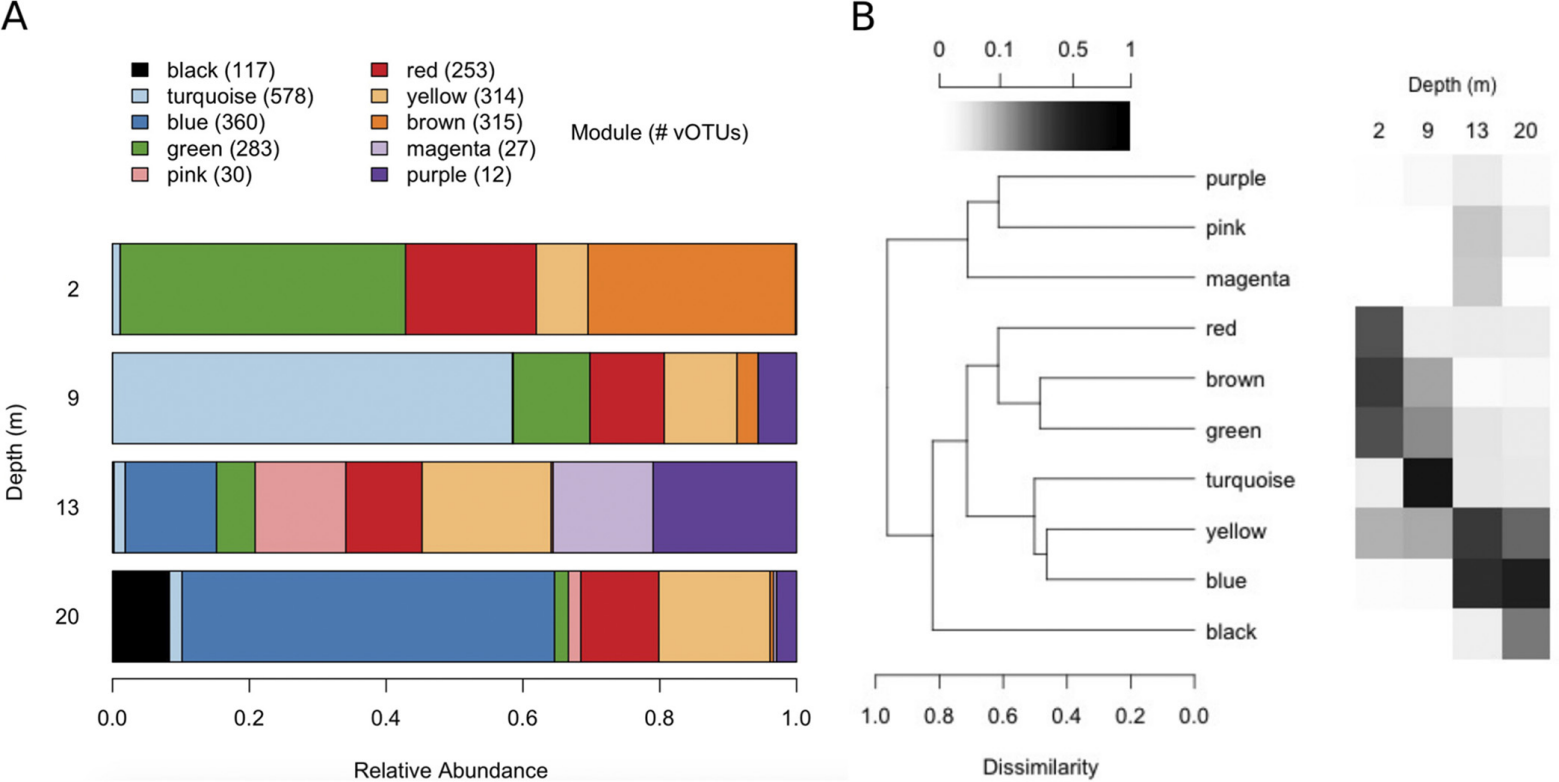
(A) Distribution of WGCNA modules by depth. The number of vOTUs assigned to each module is given in parentheses. Relative abundance was calculated using the vOTU table, normalized by sequence length. (B) Modules clustered using Bray-Curtis distance on KEGG functional annotations. A heat map shows the proportion of contigs at each depth assigned to that module. (Note the nonlinear scale for the gradient.)

WGCNA detected 10 clusters of co-occurring vOTUs, called “modules”, which contained 12 to 578 vOTUs per module and were assigned a color name for identification. Some modules were restricted to a single depth, while others appeared characteristic of water either above or below the halocline or were found at all depths (Fig. 6A). Individual modules consisted of vOTUs from a mix of virus families (Fig. 3B). The black module was unusual in that a single vOTU, vsNODE54_01, identified as a member of the *Siphoviridae*, accounted for 23% of reads recruited to contigs in this module.

### Genetic potential of viral community.

We identified 61,429 open reading frames (ORFs) in our vOTUs, of which 9.7% could be assigned a function with the KEGG database (29) (Table S2) and 24% using PFAM (30). To compare the functional capacities of modules, we calculated the proportion of vOTUs containing a given annotated function (defined as unique KO numbers from KEGG) and applied clustering (Fig. 6B), similar to the analysis of (31). Modules clustered by the depths where they predominated. The black module, found almost exclusively at 20 m, clustered apart, while the turquoise module, which dominated at 9 m, clustered with the marine modules blue and yellow. Inspection showed that freshwater modules, indicated in brown, red, and green, were impoverished relative to marine and turquoise modules in genes coding for DNA polymerase A, T7 primase/helicase, and ribonucleotide reductase NrdA/NrdE (Fig. S2), which together are characteristic of podovirus replisomes (32).

Putative AMGs were identified that met the criterion of possessing two flanking viral hallmark genes (33). The only photosynthesis-related AMG detected was *petF*, encoding ferredoxin. Four vOTUs carried *petF*, of which two, both *Phycodnaviridae*, met our AMG criteria. One accounted for 12.8% of reads in the pink module, while the other belonged to the turquoise module. *petF* was also found in a *Myoviridae* vOTU in the turquoise module, but had only a single flanking viral hallmark gene.

Other metabolic genes were examined from selected abundant vOTUs. The dominant vOTU of the black module, vsNODE54_01, included a number of DNA methylase genes and genes involved in carbohydrate and protein metabolism (Fig. S4A), while NODE170_03, a *Caudovirales* phage assigned to the magenta module, which dominated at 13 m, encoded a phospholipase and a metalloproteinase (Fig. S4B). While flanking viral genes were not observed for these metabolic genes, they may still be AMGs or play some other role in infection or virion production.

NODE14_04 accounted for 3% of reads in the pink module, and was our second longest NCLDV contig (55,686 bp), with 81 predicted ORFs (Fig. S4C). Its *Phycodnaviridae* identity was confirmed by the presence of a core NCLDV gene, annotated by Swiss-Prot (34) database as coding for the major capsid protein of Paramecium bursaria Chlorella virus 1 (31.8% identity) and by the NCBI nucleotide database to Micromonas pusilla virus SP1 (84.0% identity). Eighteen other ORFs had top hits to the giant virus class *Megaviricetes*, and one returned high similarity in Swiss-Prot database to a mimivirus protein. While most hits were to hypothetical proteins, some had replication and transcriptional functions or were related to host recognition and binding functions, such as LysM (lysin motif), and PAP2 (phosphatidic acid phosphatase type 2).

Three genes in NODE14_04 were flagged as potential AMGs: one orthologous to a fragment of Ycf2, a chloroplastidic ATPase of unknown function (35); a patatin-like phospholipase protein; and a putative gene for a prolyl 4-hydroxylase.

### Uncultivated viral genomes.

**FIG 7:**
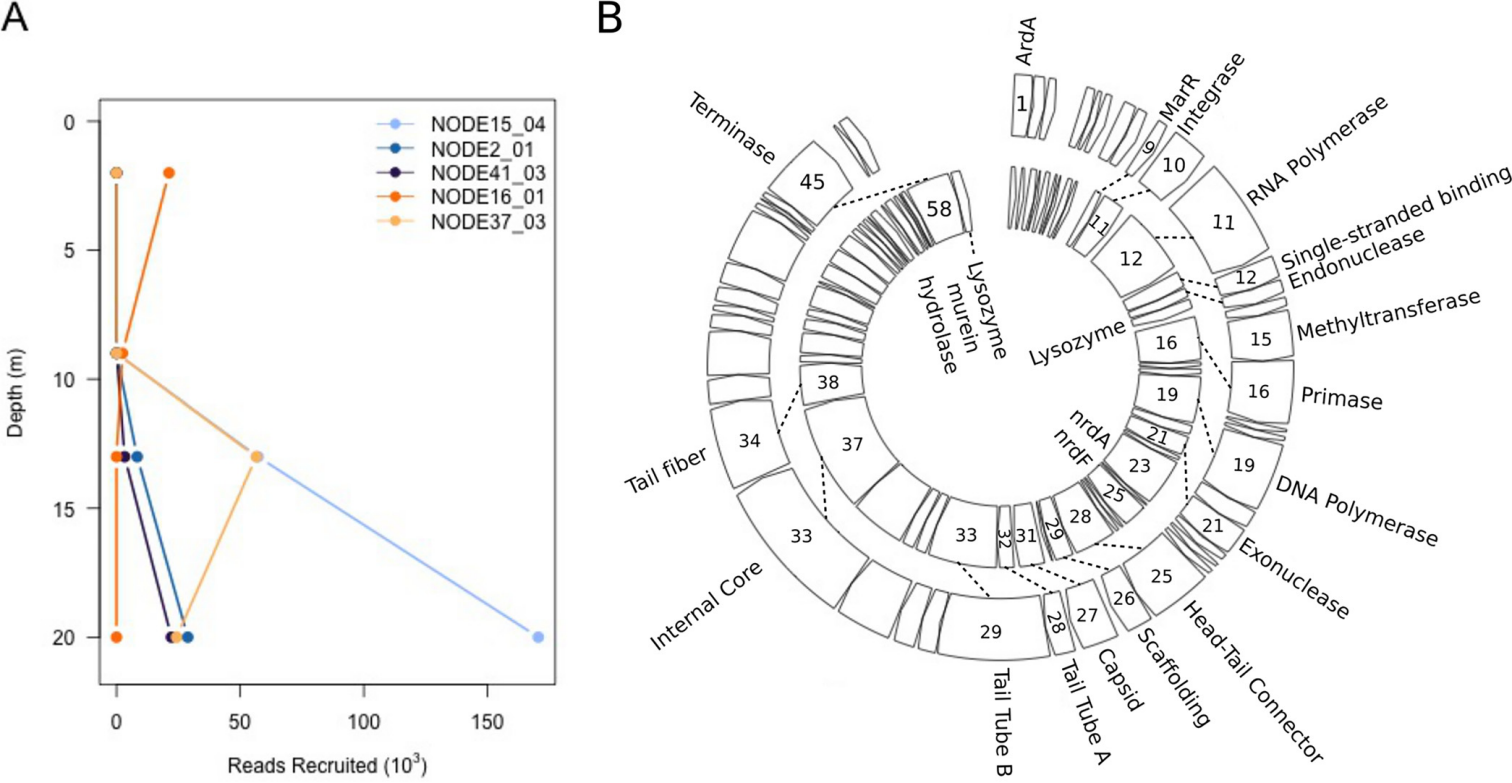
(A) Viral reads recruited to selected uncultivated viral genomes (UViGs) by depth; (B) genome organization of UViG NODE41_03 (outer ring) compared to a typical *Pelagibacter* phage, HTVC019P (inner ring), showing core genes for this viral genus. All coding is on the positive strand. Shared open reading frames (ORFs) are shown by dashed lines. The number of each ORF is shown within the arrow. A discussion of the genes, including abbreviations, may be found in the text.

We identified 15 vOTUs as potential uncultivated viral genomes (UViGs), using as criteria (i) genomes characterized as circular by VirSorter (23), (ii) those >30 kb long (the lower range for *Caudovirales* [36]), and (iii) those containing at least one viral hallmark gene (Table S3). Most belonged to the three *Caudovirales* families. One UViG ~111 kb long was identified as *Phycodnaviridae* based on its DNA polymerase B gene; however, it lacked marker genes typical for this group. We plotted the number of reads recruited to five putative UViGs that are representative of the distinctive patterns of depth distribution we observed (Fig. 7A). The majority were found only in marine layers (i.e., 13 and 20 m), while two were found exclusively in the surface, and one was found in the surface and halocline. Between 10 and 47% of ORFs in these UViGs could be annotated as encoding non-hypothetical proteins (Table S3).

The UViG NODE41_03 shares 12 core genes, and several other conserved genes with the *HTVC019virus* genus of *Pelagibacter* phages (37) (Fig. 7B). Most genomes of this genus in the NCBI RefSeq database appear linear, except for strain HTVC019, which has a 162-bp region repeated at the beginning and end of the genome sequence. Our UViG has a 55-bp repeat. The circularity of this genome remains to be verified by experimental means.

NODE41_03 is missing a lysozyme gene found in most pelagiphages; the ORF at this location has 43% identity to the closest lysozyme in NCBI. Its shorter length suggests that insertion of a methyltransferase gene truncated the original gene, rendering it nonfunctional. NODE41_03 is also missing the ribonucleotide reductase genes that precede the cassette of structural genes in most, but not all, genus members. Finally, it encodes the MarR transcriptional regulator, which has been found in one other pelagiphage genome from marine surface waters.

### Distribution of functional genes by depth.

**FIG 8:**
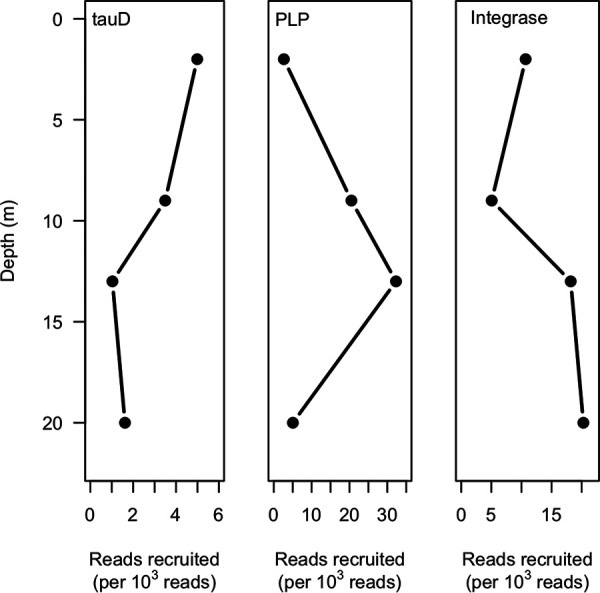
Relative abundance of reads recruited to vOTUs carrying the *tauD*, patatin-like phospholipase (PLP), and integrase functional genes mapped by depth.

We selected three functional genes and mapped the vertical distribution of viral reads recruited to vOTUs containing them. The gene *tauD*, required to use taurine as a sulfur source, is expressed under conditions of sulfate starvation (38). Reads recruited to vOTUs containing *tauD* had the highest relative abundance in freshwater and halocline layers (Fig. 8, left panel): *tauD* was particularly enriched in red and brown modules (Fig. S5D) and absent from modules associated with the 13-m depth (magenta, pink, and purple). In two vOTUs, both belonging to the turquoise module, identification as an AMG was supported by flanking viral genes. *tauD* was mainly found in *Myoviridae* vOTUs (65% of reads recruited), with smaller numbers of *Siphoviridae* and unclassified *Caudovirales*. No *Podoviridae* vOTUs encoded *tauD.* Top hits with BLASTp were to uncultured freshwater *Caudovirales* phages, for 86% and 37% of reads at 2 m and 9 m, respectively, while *tauD* genes at 13 m and 20 m had top hits to diverse prokaryote phyla.

Patatin-like phospholipase (PLP) is an enzyme that cleaves fatty acids. Reads recruited to vOTUs containing PLP genes peaked at 13 m (Fig. 8, middle panel) and were enriched in modules associated with this depth (Fig. S5E). PLP was found mainly in vOTUs assigned to *Phycodnaviridae* (92% of reads recruited), with the remainder either unclassified *Caudovirales* or not assigned. PLP genes from *Phycodnaviridae* vOTUs had >50% identity to *Phycodnaviridae* genomes in GenBank, while PLP genes from *Caudovirales* vOTUs yielded hits to bacteria only.

Integrase plays a role in the phage lysogenic life cycle, and its presence has been used to indicate the capacity for lysogeny, even in free virions (39), such as those our sampling method targeted. Integrase genes were found in vOTUs assigned to all families in the order *Caudovirales*, but not *Phycodnaviridae*. The proportion of reads recruited to vOTUs carrying integrase genes was highest in marine layers and lowest at the halocline (Fig. 8, right panel), and the integrase gene was enriched in the yellow module (Fig. S5F).

## DISCUSSION

### Milne Fiord epishelf lake ecosystem.

While the seasonal nature of freshwater inputs to MEL forces us to treat interannual comparisons cautiously, the halocline has risen by ~10 m since it was first measured 40 years ago, and its depth of 8 m in July 2016 was equal to the previous shallowest record in 2013 (7), indicating continuing deterioration of the ice shelf. In this changing physical context, the microbial community of MEL has shown both stability and change. Pigment analysis, supported by 18S rRNA genes, indicates a stratified phytoplankton community consistent with previous years, including the presence of small prasinophyte phytoplankton below the halocline (12, 13). The multiyear declining trend we observed in cyanobacterial abundance has been attributed to increasing marine influence in MEL (13), since cyanobacteria have very low abundance in the Arctic Ocean (40). While high variability did not allow us to discern a difference in abundance by depth, 16S rRNA gene and pigment data suggest that cyanobacteria may be more abundant at 2 m, perhaps associated with the plume of the input stream in Neige Bay, given the ubiquitous presence of these phototrophs in polar freshwaters (40).

Despite its isolation and precarious existence over a decadal scale, the MEL viral community is abundant, diverse, and distinct from that of the underlying fjord waters. Abundance of virus-like particles fell within the typical range for ice-based environments (41) and was higher than comparable stratified polar environments (16, 18), suggesting that viruses are abundant enough to impact microbial production and diversity in MEL.

### Viral diversity.

Since we analyzed sequences from DNA, our methodology excluded RNA viruses, which have a role in infecting eukaryotes in marine environments (42), while our analytical pipeline is biased toward *Caudovirales* bacteriophages and excludes single-stranded DNA (ssDNA) viruses (43), which predominate in some freshwater polar environments (44). Prefiltration of our water samples at 0.22 μm also causes us to miss a portion of the viral diversity (45), in particular by removing some nucleocytoplasmic large DNA viruses (NCLDVs), including *Mimiviridae*, and larger *Phycodnaviridae* (46). Prefiltration can result in underrepresentation of lysogenic viruses, which may be found as prophages within host cells; however, given that a previous study in a polar lake in summer found a lytic lifestyle predominated (16), we consider that the majority of the dsDNA viral diversity was likely present as free virions.

Identification of viral taxa is challenging because of the lack of conserved genes, high sequence divergence, and limited reference genomes (47). In general, similarity to reference genomes is not an effective means of identifying viruses in freshwater environments, which tend to be highly novel (48, 49), especially in remote environments such as the High Arctic, which have received comparatively little sampling effort (18). To analyze viral diversity, we therefore used operationally defined taxonomic units and ecology-based network analysis, as well as an automated pipeline that classified vOTUs to the family level. Identification of *Phycodnaviridae* requires particular caution, since standard databases contain many mimiviruses misclassified as phycodnaviruses (50).

Our results in Milne Fiord agreed with a study of a high-latitude meromictic lake, which found a marked distinction between viral diversity above and below the halocline (18). We hypothesize that viral distributions reflect the distribution of their hosts, whose pronounced vertical stratification in MEL is known from previous observations (13) and was confirmed in the present study (Fig. 2B and C; see Fig. S2 in the supplemental material). Host-virus connectivity of bacteriophages can be analyzed using techniques such as screening viral sequences against databases of CRISPR-Cas spacers from the bacterial antivirus defense system (51); however, such an analysis was beyond the scope of the present study.

The small fraction of vOTUs detected both above and below the halocline (i.e., euryhaline) highlights the uniqueness of the MEL ecosystem. While the viral community at the halocline resembled the freshwater layer above (Fig. 5A and B), its high proportion of unique vOTUs (36%) indicates that it offers specialized niches for viruses and their hosts. In contrast, although pigment profiles at 13 and 20 m suggested that the two depths have different phytoplankton communities, 85% of marine vOTUs, including both phage families and *Phycodnaviridae*, were common to both marine sampling depths, suggesting commonality in terms of phytoplankton and bacterial hosts. Lower taxonomic diversity in marine layers may reflect fewer niches for specialized taxa. *Phycodnaviridae* was overrepresented among euryhaline vOTUs, which could either reflect a broader host range or a specific host with a euryhaline distribution—for example the chlorophyte *Radicarteria* clade (13). Virophages were detected at 2 m in low abundance, while only a single vOTU was detected belonging to their viral host, *Mimiviridae*. Their low relative abundances may be an artifact of our prefiltration step, since a greater diversity of virophages and *Mimiviridae* are found in size fractions of >0.45 μm, probably because of *Mimiviridae*’s large size (45).

Because practically every environmental parameter was confounded with depth in our sampling (Table S1), our sample design did not allow us to disentangle the roles of individual parameters that define niches for viral taxa. However, WGCNA showed that these niches varied in location and breadth. For example, the turquoise module contains vOTUs mostly or entirely restricted to the halocline and is also richest in vOTUs, contributing to high viral diversity at this depth (Fig. 6A). In contrast, the black module is restricted to 20 m and associated with a smaller number of abundant vOTUs. Finally, the 13-m depth is distinguished by three specific modules, pink, magenta, and purple, which are likely associated with the peak of prasinophytes, as all three contain a higher proportion of vOTUs assigned to *Phycodnaviridae* (59, 33, and 87%, respectively) (Fig. 3B), which infects eukaryote algae. *Phycodnaviridae* contribute to lower diversity at 13 m, because our methods capture lower genetic diversity for this family, as discussed above. Additionally, limitations of our sequence assembly result in incomplete genome recovery, since the median length of *Phycodnaviridae* contigs was 16 kb, while genomes in this family are typically hundreds of kilobases (52). This may bias diversity estimates in unpredictable ways.

Annotation revealed that WGCNA modules reflect the genetic repertoire of virus communities, with modules clustering by gene content and depth (Fig. 6B). Similarities between the turquoise module and blue and yellow “marine” modules suggest that marine influence at the halocline is reflected in the viral community. These similarities are driven by a greater fraction of *Podoviridae*-type replication genes, corresponding to a greater abundance of this family in these modules (Fig. S5A to C). On the other hand, the turquoise module is impoverished in integrase genes (Fig. S5F), detected at lower relative abundance at the halocline (Fig. 8), indicating that a key difference between turquoise and marine modules may be a lower prevalence of lysogeny. This may correspond to the significant peak of bacterial abundance detected with FCM at 9 m, since higher bacterial production has been shown to favor lysis over lysogeny in a marine Arctic environment (53), possibly because higher host abundance and turnover favor propagation of lytic viruses.

### Assemblage of complete viral genomes.

While most of our vOTUs are genome fragments that may lack important information to elucidate taxonomy and function, we recovered 15 UViGs whose completeness could be confidently attested because of their circular character. While linear genomes are also common in viruses, we will not discuss them further because of the challenge of determining whether sequence is missing from the ends of a linear genome. As these viruses were present at abundances allowing sufficient sequence coverage, they likely represent ecologically relevant community members. UViGs varied in their distributions, although none were euryhaline or were restricted to the halocline (Fig. 7A; Table S3). Only one was a putative NCLDV, reflecting the greater coverage needed to retrieve the longer genomes of this group.

Most UViGs identified had little similarity to reference genomes in NCBI RefSeq, indicating they may be novel taxa. The exception was NODE41_03, with a strong resemblance to a *Pelagibacter* phage (formerly a member of *Podoviridae*, now *Autographiviridae*) (37). Typical for this family, the UViG contains its own RNA polymerase gene and an integrase gene, indicating that it is capable of lysogeny. Its putative host, the abundant marine bacterioplankton *Pelagibacter*, was found at high frequency in our 16S rRNA data and may belong to SAR11 clade I, whose vertical distribution resembled that of NODE41_03 (Fig. S2). In 2011, *Pelagibacter* comprised only ~1% of bacterial 16S amplicons in the MEL seawater layer (13), a low relative abundance that may be attributable to underestimation in amplicon-based surveys, since streamlined *Pelagibacter* genomes contain only one 16S rRNA gene (54).

### Auxiliary metabolic genes.

Viral ecologists increasingly recognize the ability of viruses to manipulate host function by carrying AMGs (17). To identify putative AMGs *in silico*, we inspected vOTUs whose abundance suggested they were ecologically important and identified genes with metabolic functions, including some that met the criteria of AMGs. One such gene in the *Phycodnaviridae* vOTU NODE14_04 encoded PLP, which has diverse proposed functions, including programmed cell death response to pathogens in higher plants (55), phospholipid scavenging, and penetrating the host cell in parasitic bacteria (56). Virally encoded PLP could thus support cell signaling or phospholipid metabolism of the host during infection. Alternately, it could facilitate cell wall digestion during the initial viral attachment and attack, a strategy observed in *Phycodnaviridae* chloroviruses (57), or scavenge host phospholipids for build-up of the *Phycodnaviridae* particle lipid bilayer structure (52). Detection of PLP genes in almost 10% of *Phycodnaviridae* vOTUs, its near absence in other families, and BLAST hits to *Phycodnaviridae* reference genomes all suggest a role in the unique propagation of this family.

A second AMG, *tauD*, could be expected to enhance host production under conditions of sulfur starvation. *tauD* is widely distributed among both bacterial phyla and viromes, in habitats that include freshwater, marine, and soil (58, 59). In MEL, we detected it at all depths, but at a higher frequency in freshwater and halocline, agreeing with previous findings that *tauA* in the same operon was most prevalent in microbial metagenomes from the surface waters of a stratified Arctic lake (31). Although sulfur has not been measured in Milne Fiord, this element is generally in more limited supply in freshwater than in marine environments (60), and water column sulfate has been reported to have high temporal variability in at least one Arctic lake (61).

Photosynthesis-related genes were among the earliest identified AMGs (62). The presence of only one photosynthesis-related AMG, *petF*, in MEL and the absence of *psbA*, which is considered part of the core genome of cyanomyoviruses (63), are in accordance with the low abundance of cyanobacterial hosts detected by FCM, pigment, and SSU rRNA gene analyses, although the absence of viral *psbA* does not rule out the presence of cyanophages, since some lack this gene, including one from a subarctic lake (64). Previous studies have also found higher representation of cyanophages in the size fraction of >0.45 μm, which was not captured by our sampling (45).

### Conclusions.

Our results highlight the uniqueness of the MEL viral community compared to the marine fiord water and, particularly, the halocline community, which offers niches for viruses and hosts not found in either freshwater or marine layers. High bacterial abundance coupled with a possible prevalence of lytic lifestyle at this depth suggests that viruses have an important role in biomass turnover. Identification of AMGs in MEL suggests that viruses also impact the community indirectly by influencing host metabolism.

Our results offer insights into how highly stratified environments, which can also include meromictic lakes and salt-wedge estuaries, may be affected by loss of their stratification. Some changes already apparent in the MEL ecosystem, such as the decline in cyanobacteria, may be related to the progressive shoaling of the halocline. The distinct MEL viral community draws attention to the microbial diversity of ice-dependent ecosystems in the LIA and their vulnerability to climate change.

## MATERIALS AND METHODS

### Sample collection and processing.

Water samples were collected using a 7-L Limnos water sampler (Limnos.pl, Komorów, Poland) on 17 July 2016 by boring three holes through the ~87-cm-thick ice cover. Temperature and conductivity profiling of the water column was done using a YSI 600QS probe (YSI, Yellow Spring, OH) and an RBR Concerto CTD (RBR, Ltd., Ottawa, Canada). Water was transferred into polyethylene containers cleaned with 2% (vol/vol) Contrad liquid detergent (Decon, King of Prussia, PA) and 10% (vol/vol) HCl and rinsed with water from the corresponding sample depth. Samples were kept cool and in the dark during transport to a field laboratory and processed within 6 h.

Samples for total phosphorus, total nitrogen, dissolved organic carbon, and dissolved inorganic carbon were obtained by pooling water from the triplicate samples for each depth, stored at 4°C in the dark, and analyzed as described in reference 65. Subsamples for FCM were preserved at a final concentration of 0.5% (vol/vol) glutaraldehyde. Pigment sampling and analysis by high-pressure liquid chromatography (HPLC) are described in reference 13. HPLC samples were frozen immediately at −20°C. Viral DNA was collected by filtering 1 L of water at a pore size of 0.02 μm, as described in reference 18. Briefly, we removed most cellular organisms by filtering water through a 0.22-μm capsule filter (Millipore Sterivex-GV) using a peristaltic pump and then collected virus-sized particles on 25-mm-diameter 0.02-μm-pore-size Anotop aluminum oxide filters (Whatman). In this ultraoligotrophic environment, pumping was efficient, except for one replicate from 20 m (which yielded anomalous pigment results), which clogged, requiring us to stopped filtration after 0.82 L. Filters were immediately frozen at −20°C. After transport to Université Laval, Québec City, they were stored at −80°C until extraction.

### DNA extraction and sequencing.

We extracted total nucleic acids directly from filters using the MasterPure complete DNA and RNA purification kit (Lucigen, Middleton, WI) and the backflushing technique (66). Subsequent steps used DNA-specific kits. We prepared libraries for each filter with 10 ng DNA using the NEB Next Ultra II library preparation kit (New England Biolabs, Ipswich, MA). Paired-end sequencing was performed on a HiSeq 2500 system (Illumina) at the McGill University and Génome Quebec Innovation Centre (Montreal, Canada), yielding 9 to 20 million reads (of 125 bp) per library (see Table S4 in the supplemental material).

### Sequence processing.

Sequence processing used a previously described pipeline (18) to remove low-quality reads, trim low-quality nucleotides, assemble contigs, identify viral sequences, cluster contigs into vOTUs using the threshold of 95% identity over 85% of their length, map quality-controlled reads to vOTUs, and merge files. Only viral contigs of ≥10 kb were clustered into vOTUs, and we used the longest contig of each vOTU as a representative sequence. For distance-based and network analyses, we normalized counts by vOTU sequence length using Read2Ref Mapper v.1.1.0 (67), to account for higher recruitment to longer vOTUs, and applied Hellinger and log transformations. Rare vOTUs (<1% relative abundance or present in only one replicate of a sample) were removed from the normalized table, as recommended for noisy high-throughput sequencing data sets (68). An unprocessed vOTU table, keeping rare vOTUs, was used for diversity and presence-absence analyses. We determined putative taxonomic identity to order or family using VPF-Class (69), with classification files provided by the author and a membership ratio cutoff of 0.5.

We predicted ORFs using Prodigal v.2.6.3 with the meta option (70) and screened them against the KEGG database using the online platform GhostKOALA (71) as well as using DRAM-v (33) with default parameters. DRAM-v identifies potential AMGs by searching sequences against multiple databases, including Pfam (30), with a threshold of >35% coverage and an E value of <10^−15^, and a BLAST-type search against all viral proteins in NCBI RefSeq. ORFs of NCLDVs were further annotated by searches against Swiss-Prot (34), TIGRFAM (72), Gene3D (73), and Superfamily (74) using a threshold E value of <10^−10^ for all databases. Contigs with potential AMGs of interest were manually verified for flanking viral genes and by a BLASTP search of the gene sequence.

### Statistical analysis.

We tested for significant difference in alpha diversity using a Kruskal-Wallis rank sum test with *post hoc* Dunn’s test between depths with a Bonferroni correction. Clustering analysis was performed on normalized vOTU abundance data using Bray-Curtis distance with the R package vegan (75). We performed WGCNA with the R package WGCNA (76). Modules were clustered by gene functions using Bray-Curtis distance.

### Small subunit rRNA genes.

We used the phyloFlash pipeline v3.4 (77) to screen all quality-controlled reads for SSU rRNA genes by mapping against the SILVA SSU Ref NR 99 database 138.1 (78) with default settings and the parameter “-almosteverything.” The last-common-ancestor consensus of top hits was used to report an approximate taxonomic affiliation.

### Data availability.

Raw sequences are available in NCBI SRA database under BioProject no. PRJNA781624. Viral contigs and a vOTU table are available at https://github.com/LabViDEL/Milne-Fiord-2016.
