# Supplementary material for: Climate-Endangered Arctic Epishelf Lake Harbors Viral Assemblages with Distinct Genetic Repertoires
Source: Appl Environ Microbiol. 2022 Aug 25;88(17):e00228-22. doi: 10.1128/aem.00228-22 (PMC9469726; doi:10.1128/aem.00228-22)
Supplement: Supplemental file 1 — Tables S1, S3, and S4 and Fig. S1 to S5. Download aem.00228-22-s0001.pdf, PDF file, 2.4 MB [file aem.00228-22-s0001.pdf]

Supplementary Table S1. Physicochemical properties of the water column and the correlation (Pearson correlation analysis) of these variables with sample depth.

Depth (m)	Irradiance (µmol $m^{-2}s^{-1}$)	pH	Temperature (°C)	Conductivity (mS m^{-1})	Oxygen (%)	Dissolved Organic Carbon (mg l^{-1})	Dissolved Inorganic Carbon (mg l^{-1})	Total Nitrogen (µg l^{-1})	Total Phosphorus (µg l^{-1})
2	153	6.96	1.07	0.40	114.9	0.41	3.0	35.84	20.40
9	1.55	7.10	0.20	9.21	113.9	0.47	6.5	74.04	19.54
13	–	7.26	-0.93	41.84	90.4	0.95	21.4	235.89	5.00
20	–	7.75	-1.15	43.07	86.9	1.16	22.5	292.83	12.01
**Correlation with Depth**	-0.8	0.96	-0.95	0.9	-0.89	0.93	0.91	0.94	-0.64

Table S3. Characteristics of putative Uncultivated Viral Genomes (UViGs), which have been identified as circular by VirSorter. Viral Family has been determined by VPF-Class, and Module is determined by WGCNA as described in the text. '*' indicates UViGs that are shown in Figure 7A.

Contig identifier	Viral Family	Module	Length (bp)	Open Reading Frames	Non-hypothetical proteins	Total Reads recruited to vOTU	Distribution
NODE51_04	Myoviridae	Yellow	37 365	50	5	43 595	Marine
NODE34_03	Myoviridae	Yellow	42 107	51	10	97 385	Marine
NODE30_02	Myoviridae	Brown	39 941	58	7	27 174	Surface-Halocline
NODE684_01	Podoviridae	Blue	31 966	44	13	139 816	Marine
NODE62_02	Siphoviridae	Yellow	31 876	47	8	40 826	Marine
NODE42_03	Myoviridae	Blue	38 599	52	16	30 391	Marine
NODE72_04*	Podoviridae	Green	34 569	48	9	38 620	Surface-Halocline
NODE2_01*	Phycodnaviridae	Blue	110 748	116	15	37 163	Marine
NODE24_03	Podoviridae	Blue	55 116	76	13	45 917	Marine
NODE41_03*	Podoviridae	Blue	39 547	47	20	25 472	Marine
NODE37_03*	Podoviridae	Yellow	36 431	46	9	80 750	Marine
NODE16_01*	Siphoviridae	Brown	48 628	69	13	23 627	Surface-Halocline
NODE41_02	Podoviridae	Blue	38 968	36	17	142 288	Marine
NODE15_04*	Podoviridae	Blue	61 246	72	15	227 725	Marine
NODE12_03	Podoviridae	Yellow	68 348	88	17	41 845	Marine

Table S4. Number of reads obtained per library.

Depth (m)	Replicate	Number of reads
2	A	19 666 448
	B	18 695 438
	C	15 932 910
9	A	20 104 378
	B	9 513 168
	C	15 110 742
13	A	19 860 086
	B	18 470 662
	C	19 974 192
20	A	14 687 992
	B	18 000 044
	C	18 210 582

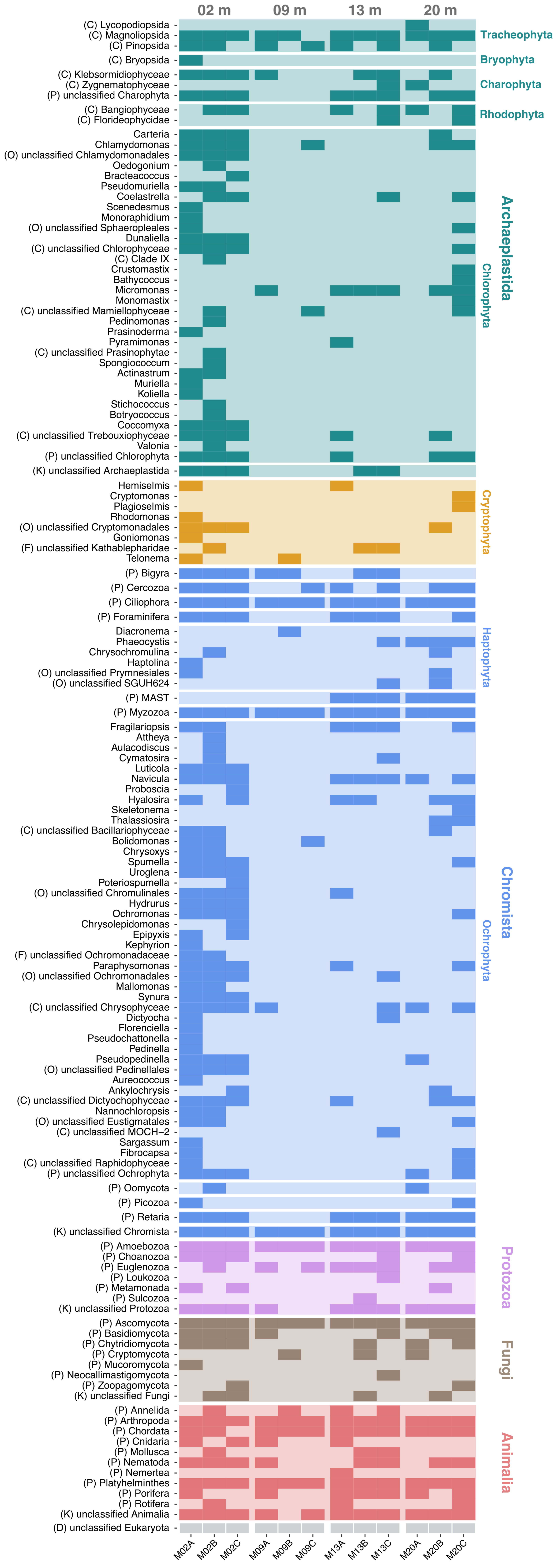

Figure S1. Presence/Absence of eukaryote taxa (shown as dark/pale fill) detected by 18S rRNA genes in replicate samples from four depths. Named taxa are genera, unless indicated by parenthesis. K = Kingdom; P = Phylum; C = Class; O = Order; F = Family.

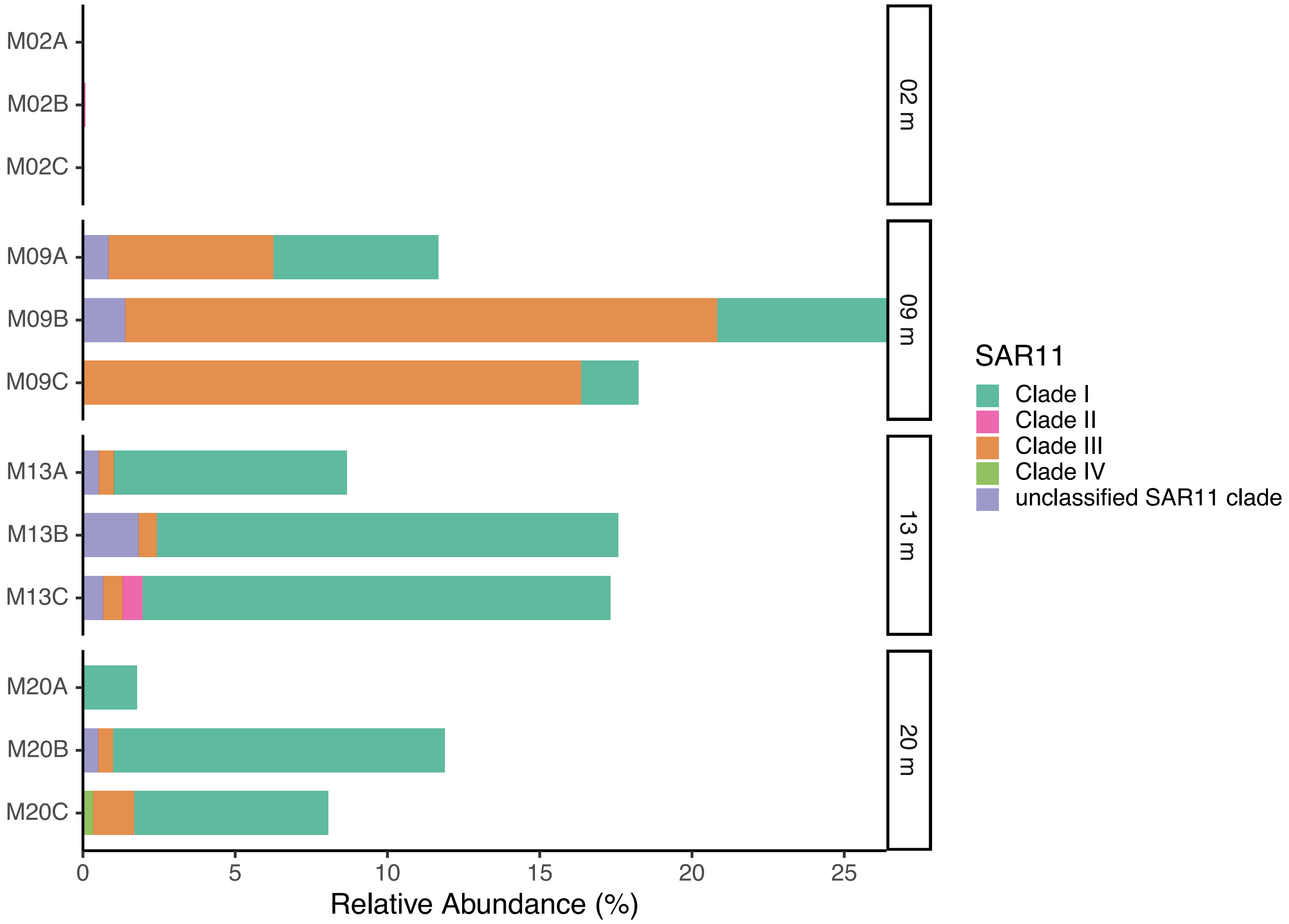

Figure S2. *Pelagibacter* (SAR11) clades detected by 16S rRNA genes in replicate samples from four depths.

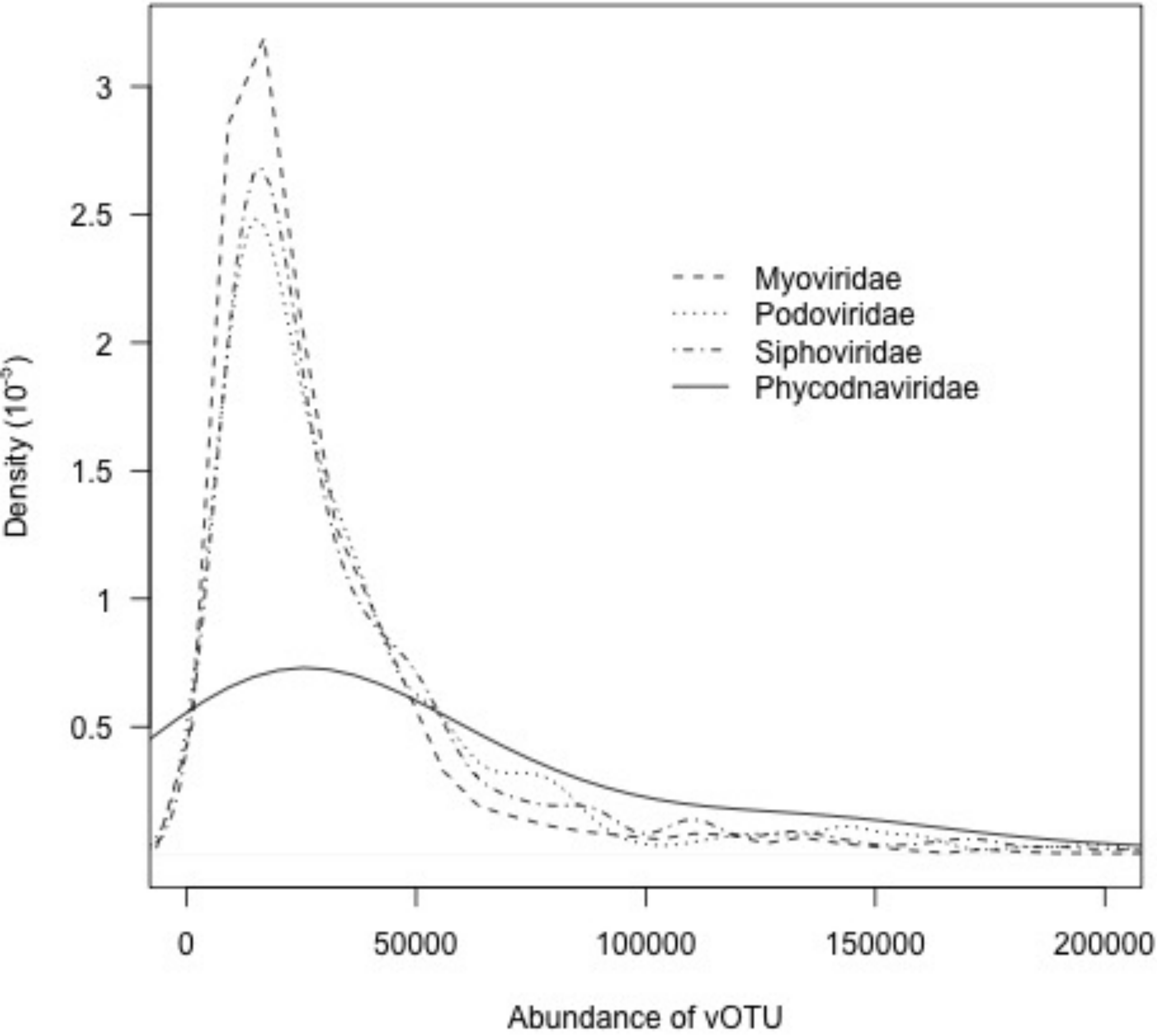

Figure S3. Comparison of vOTU abundance and frequency between virus families. A flatter peak indicates fewer, and more abundant vOTUs. vOTUs were clustered at 95% identity over 85% of the sequence length. Gaussian kernel density estimation was plotted using a fast Fourier transform, with bandwidth value selected using Silverman's rule of thumb, such that the bandwidth was the standard deviation of the smoothing kernels. Note that x axis is truncated.

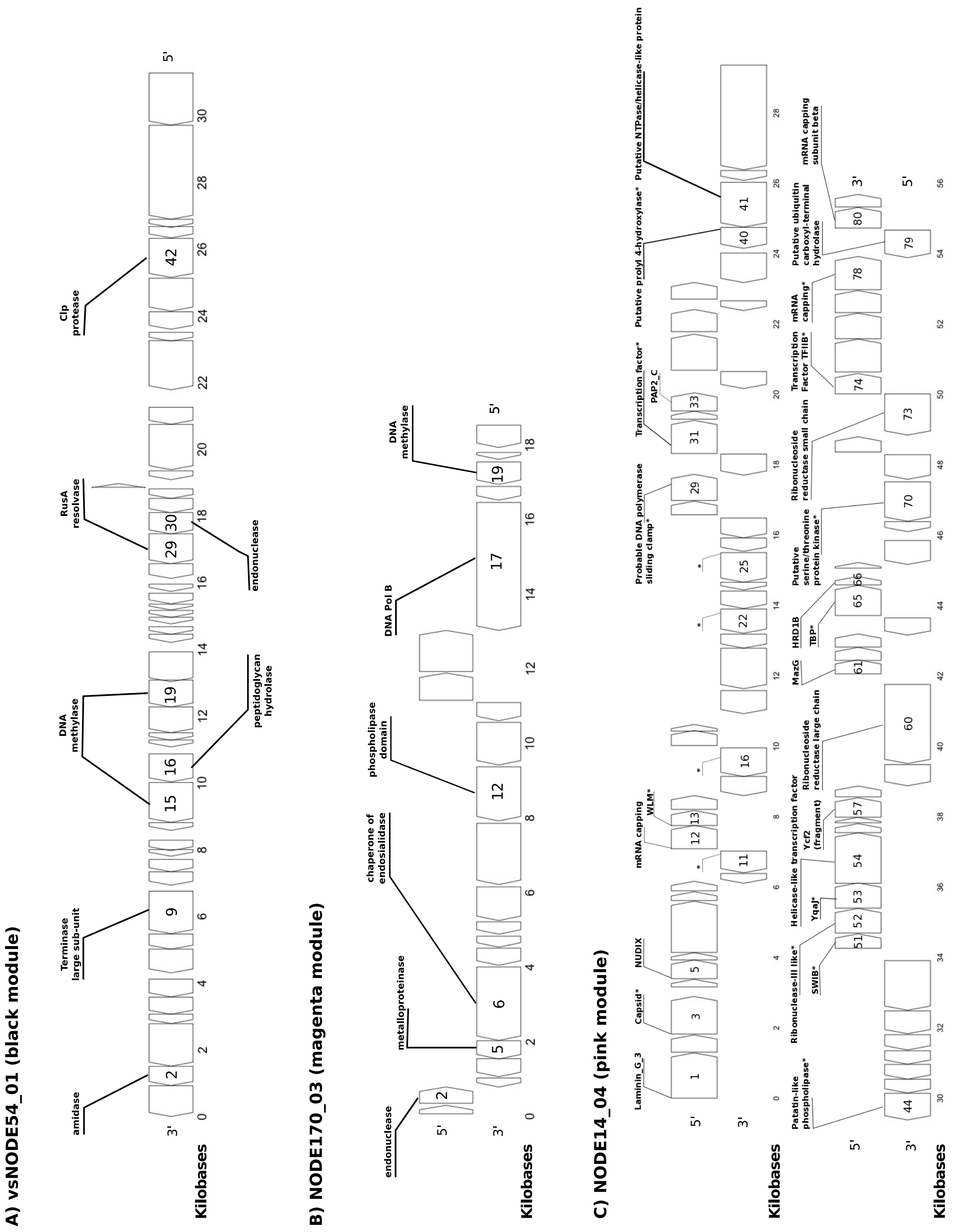

Figure S4. Gene annotation from RefSeq, KEGG, and PFAM databases of selected vOTUs with ecological significance, representing genome fragments, **(a)** vsNODE54_01, **(b)** NODE170_03, and **(c)** NODE14_04. In (c), orthologs of genes with best hit to *Phycodnaviridae* genomes in GenBank are starred (*). The numbering of each Open Reading Frame is shown within the arrow. Abbreviations: DNA Pol, DNA polymerase; NUDIX, nucleoside diphosphate linked moiety X; WLM, Wss1p-like metalloprotease; PAP2_C, Type 2 phosphatidic acid phosphatase; SWIB, SWI/SNF (Switch/Sucrose non-fermentable) complex B; TBP, TATA-box binding protein; HRD1B, HMG-CoA reductase degradation ubiquitin ligase 1B.

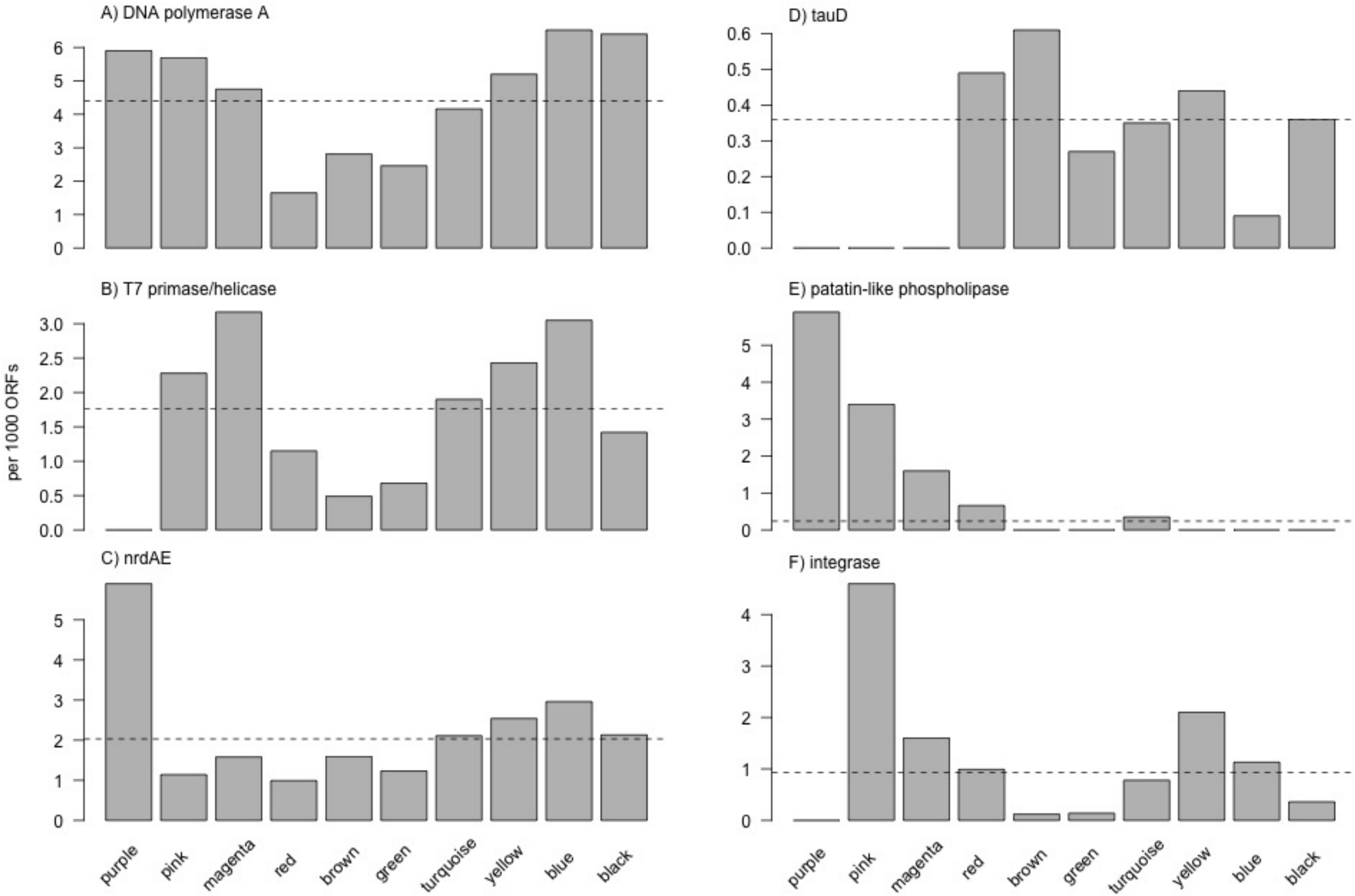

Figure S5. Fraction of Open Reading Frames (ORFs) annotated with genes for **(a)** DNA polymerase A, **(b)** T7 primase/helicase, **(c)** RNA reductase, **(d)** *tauD*, **(e)** patatin-like phospholipid, and **(f)** integrase, by WGCNA module. Dashed line represents overall fraction of ORFs (modules pooled together). Note that a lower diversity of vOTUs in the modules purple, pink, and magenta contributes to less reliable values for these modules.
